# New micro-hole zone catheter reduces residual urine and mucosal microtrauma in a lower urinary tract model

**DOI:** 10.1038/s41598-024-52505-6

**Published:** 2024-01-27

**Authors:** Brit Schrøder, Fabio Tentor, Teodora Miclăuş, Kristian Stærk, Thomas Emil Andersen, Michele Spinelli, Claudia Rendeli, Giulio Del Popolo, Per Bagi, Lene Feldskov Nielsen

**Affiliations:** 1 Coloplast A/S, Holtedam 1, 3050 Humlebaek, Denmark; 2 AML Christensen, Nivaa Strandpark 21, Nivaa, Denmark; 3 Department of Urology, Rigshospitalet, Blegdamsvej 9, 2100 Copenhagen, Denmark; 4 Department of Clinical Microbiology, Odense University Hospital, Odense, Denmark; 5 Research Unit of Clinical Microbiology, University of Southern Denmark, Odense, Denmark; 6 Spinal Unit, Niguarda Hospital, Milan, Italy; 7 Department of Pediatrics, Università Cattolica del Sacro Cuore, Rome, Italy; 8 Neuro-Urology Department, Azienda Ospedaliero-Universitaria Careggi, Florence, Italy

**Keywords:** Biological models, Bladder

## Abstract

Urinary tract infections (UTIs) are the main complication associated with clean intermittent catheterization (CIC) and are facilitated by post-void residual urine and trauma to the mucosa during voiding. The risk of UTI may be diminished by reducing the residual volumes and preventing microtrauma caused by mucosal suction through the eyelets of conventional eyelet catheters (CEC). A new micro-hole zone catheter (MHZC) was developed and tested in an ex vivo porcine lower urinary tract model and in vivo, in pigs, against a CEC. It was shown that, irrespective of the micro-hole diameter, the new catheter ensured increased flowrates and significantly lower residual volumes at the first flow-stop. Furthermore, with a micro-hole diameter of 0.4 mm, mucosal suction was virtually eliminated, regardless of the insertion depth or simulated intra-abdominal pressure mimicking sitting or standing humans. Pressure profile experiments and endoscopy studies indicated that the bladder gradually folds against the drainage tip of the new catheter, without blocking the flow, and, unlike with the CEC, sharp pressure variations and flow-stops did not occur during voiding. The MHZC outperformed the CEC in all tested scenarios and decreased residual volumes, thus potentially decreasing the risk of UTIs.

## Introduction

Clean intermittent catheterization (CIC) is the standard of care for bladder management in neurogenic or non-neurogenic lower urinary tract dysfunctions (LUTDs) of various etiologies^1–4^. Urinary tract infections (UTIs) represent the main complication associated with CIC. UTI incidence in patients practicing CIC varies widely, depending on LUTD causes, time of assessment after initiation of catheterization, patient-related parameters, as well as how such infections are defined in each study. Values ranging from as low as 2%^2^, to as high as over 70%^5^ have been reported, and incidence over 30% is not uncommon^6–9^.

Kennelly et al.^10^ recently highlighted post-void residual urine following improper catheterization, as well as uroepithelial trauma from the intermittent catheter (IC) as important risk factors for UTI. There are indications that, ideally, the post-void residual volume after catheterization should be below 6 mL^11^. In practice, however, this is not always reached, with some studies reporting that over 70% of participants had residual urine in excess of 50 mL and that even small amounts of urine in excess of the target volume may increase the risk of UTI^12–14^. Furthermore, trauma may occur during catheterization, ranging from minor mucosal tears to serious complications such as false passage, and is also associated with increased risk of developing UTIs^10,15^.

Avoiding high post-void residual volume and reducing microtrauma can be done using proper catheterization technique and hydrophilic-coated, single use ICs^10,16,17^. However, a recent study on the performance of 3 commonly used ICs in an ex vivo porcine bladder model showed that microtrauma resulting from the suction of the bladder mucosa through the 2 eyelets of the catheter is not uncommon with conventional eyelet catheters (CECs). Furthermore, the same study indicated that residual volumes at the first flow-stop were non-negligeable^18^. In practice, patients would need to reposition the IC in order to completely empty the bladder and this procedure may not be carried out properly, or could be associated with additional microtrauma. Therefore, an ideal catheter would be designed such as to reduce or even eliminate mucosal suction and to avoid flow-stops before complete voiding^19^.

While device materials, coatings, usability, and discretion of ICs have been improved in recent years, the basic drainage principle has remained the same^10,19^. In this study, using a previously developed ex vivo porcine bladder model^18^, as well as living pigs, a CEC was compared with a new multi-hole zone catheter (MHZC). Unlike the typical 2 eyelets used to drain urine in conventional ICs, the MHZC has a uniquely designed drainage zone, with over 80 micro-holes. The behavior of this new catheter was compared to that of a CEC with respect to performance and safety parameters namely flowrate, residual volume at first flow-stop, and mucosal suction. Recently, clinical data on the same new MHZCs was published by our group^20,21^. In the current work, we aim to thoroughly investigate the newly-designed MHZC prior to clinical use, varying essential device construction parameters, namely the diameter of the micro-holes, as well as simulated patient parameters, specifically catheterization in sitting and standing positions. We intended to test the performance of our devices against a CEC when draining clear, normal density urine in these different scenarios. Additionally, we aimed to gather knowledge of the mechanisms by which our device achieves its intended targets of reducing residual urine and mucosal damage. Taken together, these 3 studies complement each other, thus offering a more complete and solid image of the clinical performance, safety, and functioning of the new catheter.

## Methods

### Ex vivo model test system

The ex vivo model was prepared as described in detail elsewhere^18^. Briefly, fresh lower urinary tracts (LUTs) of male pigs were acquired from a local slaughterhouse (Glumsø Slagtehus ApS, Glumsø, Denmark), cleaned of excess fat, and used in the ex vivo set-up. The LUT was positioned on a silicone mold mimicking the pelvic floor, then placed in a custom-made tank, granting access to the urethra through an aperture. One ureter was then connected to a peristaltic pump to allow filling of the bladder. The tank was then filled with 150 mM NaCl solution and sealed. The pressure in the tank was controlled by adjusting the height of a saline water column connected to the tank. In this setup, the bladder was filled with 205 ± 5 mL of 150 mM NaCl at a flowrate of 5.5 mL/s through the ureter connected to the peristaltic pump. In this initial proof-of-concept study, only saline simulating clear urine was tested, as we sought to assess different catheter parameters under easy to control, reproducible conditions, in order to understand and tune device performance. As such, fluids simulating urine with debris, higher viscosity, or blood clots were beyond the scope of the current work.

### Catheter performance testing in the ex vivo model

Various parameters were assessed using two types of catheters in the ex vivo model. As a control, a CEC was used (SpeediCath Standard, Coloplast). This type of catheter was used in the development of the same ex vivo model^18^, where 3 different CECs were assessed. The IC chosen as a control in the current study was superior to other such devices and is thus representative of the performance and safety of the current state-of-the-art CEC design. The test catheter was the MHZC, with the new design having a drainage zone with over 80 micro-holes, instead of the 2 eyelets in the CEC. Initially, 4 MHZCs, with different micro-hole diameters (0.4 mm, 0.5 mm, 0.6 mm, and 0.7 mm) were assessed. All MHZCs had the same number of micro-holes. For subsequent investigations at different insertion depths and different simulated intra-abdominal pressures mimicking conditions in sitting and standing humans, only the catheter with the best performance of these 4 was compared to the CEC.

Catheters of Charrière size 12 (CH12) were used for both the control and the MHZCs in all the experiments conducted in the ex vivo model. For each set of investigated parameters (micro-hole diameters, insertion depths, simulated intra-abdominal pressure), each catheter underwent 5 repeat tests in the same bladder, with repeated measurements in 3 fresh porcine LUTs, to account for the biological variability. For each experiment, the catheter was inserted into the bladder through the urethra and kept in place by the operator throughout the voiding process. A scale with a bucket on it was placed underneath the ex vivo set-up and the saline emptied from the bladder was progressively collected, thus tracking the weight variation with time. The weight of the saline collected after 5 s of voiding was recorded, without stopping the flow. Based on the amount of saline drained in the first 5 s of the experiment, when, as seen in previous testing^18^, the flowrate remained stable, the flowrate was calculated by dividing the collected volume to the 5 s draining time. Subsequently, the weight of the saline was continuously measured in the collection bucket and the value at the first flow-stop was also recorded. The residual volume represented the difference between the total initial saline volume introduced in the bladder and the volume emptied into the collection bucket up to the first flow-stop. The operator held the catheter throughout the entire voiding process and recorded tactile feedback in the form of a sudden pulse of the catheter during flow-stop, which is indicative of mucosal suction^22,23^. After flow-stop, the catheter was repositioned, and this was repeated until all liquid was emptied from the bladder. The catheter was withdrawn and then the experiment was repeated.

Once the best performing MHZC was identified based on micro-hole diameter, only that test catheter was used for the subsequent experiments and compared to the control. Two parameters were varied to account for different use scenarios, i.e. insertion depth and simulated intra-abdominal pressure. Firstly, experiments were conducted at different catheter insertion depths, as users are not necessarily expected to insert the full catheter drainage zone into the bladder. As such, the impact of different insertion lengths on the parameters of interest was tested. The intra-abdominal pressure was set at 50 cmH_2_O, as it represents a worst-case scenario for a catheter user in standing position. The CEC was tested in each of the 3 porcine LUTs as the first and last of the catheterizations and used as an internal reference. A gap of 1 cm was kept between the lowest eyelet of the conventional IC and the bladder neck. After using a ruler to measure the catheter length and establish what portion of the device should be inserted, the MHZC was positioned in 3 configurations, namely: (A) with all the micro-holes in the bladder, with the lowest micro-hole kept at the bladder neck, (B) with half of the micro-holes inside the bladder, and (C) mimicking the way in which a user would insert the catheter, namely without any length measurement, the MHZC was pushed until flow started and then a little further, as per the instructions for use.

Secondly, for a given insertion depth, two values were used for the simulated intra-abdominal pressure. For this set of experiments, the entire drainage zone of the MHZC was inserted into the bladder, with the end of the drainage zone and the lowest catheter micro-hole kept at the bladder neck. Flowrate during the first 5 s and residual volume at first flow-stop were calculated and operator tactile sensation during voiding was documented, as described above. In order to test the impact of two different simulated intra-abdominal pressures on the performance of the MHZC with 0.4 mm holes, a sitting and a standing scenario were considered. A pressure of 20 cmH_2_O (≈ 19.6 mbar) was chosen to represent the sitting situation, and one of 50 cmH_2_O (≈ 49.0 mbar) represented the standing scenario^24,25^.

### Mechanistic study of the MHZC in the ex vivo model

To better understand what phenomena occur inside the LUT during catheterization, two types of investigations were carried out, namely intra- and peri-catheter endoscopic visualization, and intra-catheter pressure measurements.

Visualization of mucosal suction and of the bladder folding around the MHZC was carried out using a flexible endoscope (Special-Fiberscope, 3.0 mm × 100 cm, Fiberscope Series, Karl Storz SE & Co. KG, Tuttlingen, Germany). During catheterization on the ex vivo model, the endoscope was inserted through the urethra and into the bladder, parallel to the IC during the catheterization process. A mixture of fluorescent green and fluorescent red polyethylene beads (Cospheric LLC) of 125–150 μm, 150–180 μm, and 250–300 μm was suspended in a tween solution and injected into the bladder model via the second ureter. These beads were used to mimic sediments in the urine and to visualize flow through the eyelets in the ex vivo endoscopy experiments. Furthermore, a CH16 MHZC was used in a different experiment where the endoscope was inserted into the catheter lumen, allowing for intra-catheter visualization of any suction phenomenon. All endoscopic investigations presented here were carried out in the ex vivo model using the MHZC alone, as for the CEC the mucosal suction phenomenon was already demonstrated in the same ex vivo setting^18^.

Further understanding of the phenomena occurring during catheterization was gathered by measuring pressure variations directly inside the catheter, using a fiber optic pressure sensor (FISO-LS Fiber Optic Pressure Catheter, model 25-0706, FISO Technologies Inc., Quebec, Canada). The 300 μm wide pressure sensor recorded variations in the ± 300 mmHg pressure range, at a 1000 Hz sampling rate. The FISO pressure sensor was powered using an Evo Chassis (FISO Technologies Inc., Quebec, Canada), which also functioned as the digital interface for data transfer. 3D-printing (Vero material, J750, Stratasys, Minnesota, USA) was used to fabricate a custom-made adaptor for fixing the sensor inside the catheter. The sensor was inserted into the catheter while keeping a distance of 0.5 cm to the lowest edge of the eyelet/micro-hole most proximal to the catheter outlet. The sensor was held in place by the custom-made adaptor throughout the entire catheterization process. For all the pressure variation experiments, a CH12 MHZC with micro-hole diameter of 0.4 mm was used and tested at simulated intra-abdominal pressures of both 20 cmH_2_O and 50 cmH_2_O. As for all the other experiments, tests were repeated 5 times in each of 3 different porcine LUTs.

### In vivo animal study

To confirm the mechanistic functioning of the MHZC, studies were performed in 2 living female pigs (Landrace × Yorkshire, mix, weighing 45 kg) obtained from a pig herd (Kokkenborg, Denmark) having the highest health status as per the Danish Specific Pathogen Free system^26^. The pigs were pre-medicated with medetomidine (Cepetor 0.12 mg/kg), butorphanol (Butomidor 0.2 mg/kg), and midazolam (Midazolam 0.1 mg/kg) and moved to the operating bed once complete muscle relaxation was achieved. Subsequent anesthesia was induced and maintained on propofol. With the animal in supine position, catheterization was performed using a CH16 MHZC with 0.4 mm micro-hole diameter. A fiberoptic endoscope (HOPKINS Forward-oblique Telescope 30°, KARL STORZ, Germany) with a protective sheath (Ø = 3.5 mm) was placed inside the catheter in order to visualize the micro-holes during catheterization. To reflect the natural bladder emptying process, a 50 mL syringe with a steady flowrate of about 6 mL/s was used through the endoscope's working channel (representing an approximation of the natural flowrate during IC). A TELE PACK VET X LED (KARL STORZ, Germany) was used to record video and images. The catheterization of the second animal was done with a CH12 MHZC with 0.4 mm hole diameter and a pressure sensor mounted to investigate flowrate and mucosal suction pressure. After complete emptying of the bladder, it was subsequently filled with 200 mL saline solution. As in the ex vivo model, during bladder emptying the flowrate and intra-catheter pressure were measured and the tactile sensation perceived by the operator was documented.

Animal experiments were approved by the Danish Animal Experiments Inspectorate, license number 2019-15-0201-01626 and were carried out in accordance with the EU directive 2010/63/EU for animal experiments and the ARRIVE guidelines.

### Statistical analysis

GraphPad PRISM (version 9.1.2.) was used for statistical analysis and students t-tests (with Welch correction when appropriate) were performed to compare results for the MHZCs with those for the CEC, as well as between results obtained under different conditions with the same catheter (e.g. intra-abdominal pressure simulating sitting and standing positions). All data were parametric. When variances were significantly different, Welch corrections were performed. For mucosal suction, which was considered a qualitative result, no statistical analysis was performed. The threshold for significance was p < 0.05. Significance is represented in the figures as follows: ns = not significantly different, *p < 0.05, **p < 0.01, ***p < 0.001, and ****p < 0.0001.

## Results

### Performance of MHZC with different micro-hole diameters

**Figure 1:**
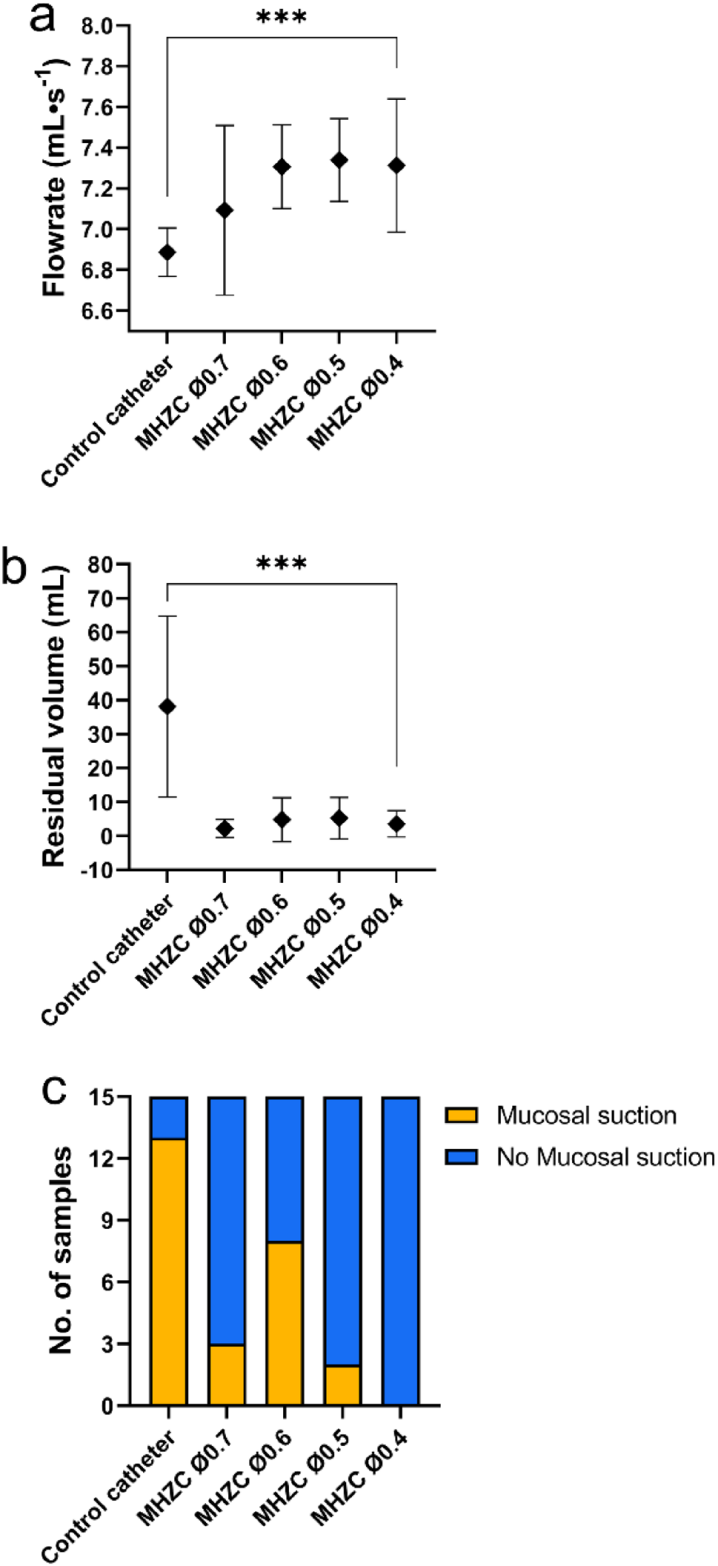
Performance of MHZCs of 0.7, 0.6, 0.5, and 0.4 mm in diameter against CEC as control, in ex vivo porcine lower urinary tract model, with comparison of (**a**) flowrate, (**b**) residual volume at the first flow-stop and (**c**) instances of mucosal suction. ***p < 0.001. CEC, conventional eyelet catheter; MHZC, micro-hole zone catheter.

The new MHZCs with 4 different micro-hole diameters (0.4 mm to 0.7 mm) were tested in the ex vivo pig LUT model against a CEC. As can be seen in Fig. 1a and b, calculated flowrates seemed higher with the new catheters, with significant differences for all 4 tested catheters, while residual volumes were about 10 times lower for all tested MHZCs compared to control. Furthermore, while the average residual volume with the CEC was close to 40 mL (38.1 ± 26.7 mL), it was below 6 mL for the MHZCs irrespective of hole diameter (2.2 ± 2.6 mL at 0.7 mm, 4.8 ± 6.5 mL at 0.6 mm, 5.2 ± 6.1 mL at 0.5 mm, and 3.6 ± 3.9 mL at 0.4 mm). Flowrates and residual volumes were comparable among the 4 different MHZCs.

Mucosal suction events were documented with all MHZCs except for the one with 0.4 mm micro-holes. The CEC had the most instances of suction (12 out of 15 catheterizations), as can be seen in Fig. 1c, while the highest number of such events (8 out of 15 catheterizations) for the test device were reported for the 0.6 mm micro-holes MHZC.

Given the above results, the MHZC with 0.4 mm micro-holes was chosen for all further experiments due to its superiority with respect to mucosal suction, while showing flowrate and residual volume similar to the other MHZCs.

### Effect of insertion depth on MHZC performance

**Figure 2:**
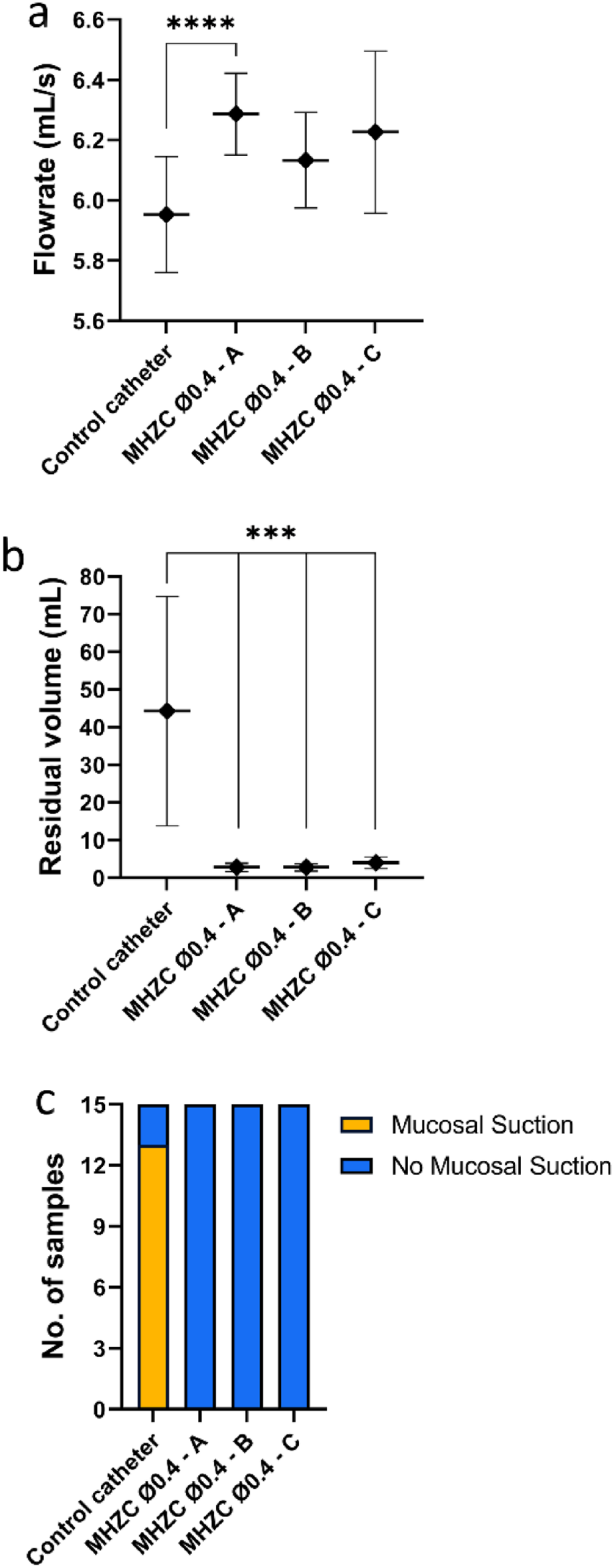
Performance of MHZC with 0.4 mm diameter inserted at different depths: A—entire drainage zone into the bladder, B—half of the drainage zone into the bladder, C—use-simulated insertion up to flow start and a bit more), compared to CEC as control, in ex vivo porcine lower urinary tract model, with comparison of: (**a**) flowrate, (**b**) residual volume at the first flow-stop, and (**c**) instances of mucosal suction. ***p < 0.001, and ****p < 0.0001. CEC, conventional eyelet catheter; MHZC, micro-hole zone catheter.

As in a real-life use scenario the draining zone of the MHZC may not be fully inserted into the bladder, experiments were conducted to account for this by testing the catheter performance at 3 different insertion depths. In 2 scenarios, the MHZC was inserted at a fixed depth, namely with the entire drainage zone in the bladder (scenario A), or with half of the drainage zone in the bladder (scenario B). As shown in Fig. 2a and b, no significant differences in flowrate or residual volume were reported between these 2 settings. In a third scenario, the user experience was mimicked as closely as possible by inserting the MHZC until the flow started, and then a bit more, as per the instructions for use. Again, no clinically significant differences in flowrate or residual volume were observed compared to scenarios A and B (Fig. 2a and b).

When compared to the CEC, all scenarios with the MHZC resulted in significantly higher mean flowrate values, with the greatest difference in the case of the fully inserted drainage zone (5.95 ± 0.19 mL/s control vs. 6.29 ± 0.14 mL/s p < 0.0001, scenario A in Fig. 2a). Residual urine was significantly lower in all 3 MHZC scenarios compared to the control (p < 0.001), with over 44.3 ± 30.5 mL for the CEC and between 2.8 ± 0.9 and 4.0 ± 1.6 mL for the MHZC (Fig. 2b). In addition, no mucosal suction events were documented with the MHZC regardless of the insertion depth, whereas suction occurred in 13 of the 15 tests conducted with the CEC (Fig. 2c).

### Effect of pressure variation on MHZC performance

With differing body postures, bladder and abdominal pressures vary in humans^27,28^, resulting in pressures between 15 and 40 cmH_2_O in sitting adults and between 20 and 50 cmH_2_O in the standing position^24,25^. To account for these variations in the current study, tests were conducted at both 20 cmH_2_O and 50 cmH_2_O, to mimic sitting and standing positions in catheter users.

**Figure 3:**
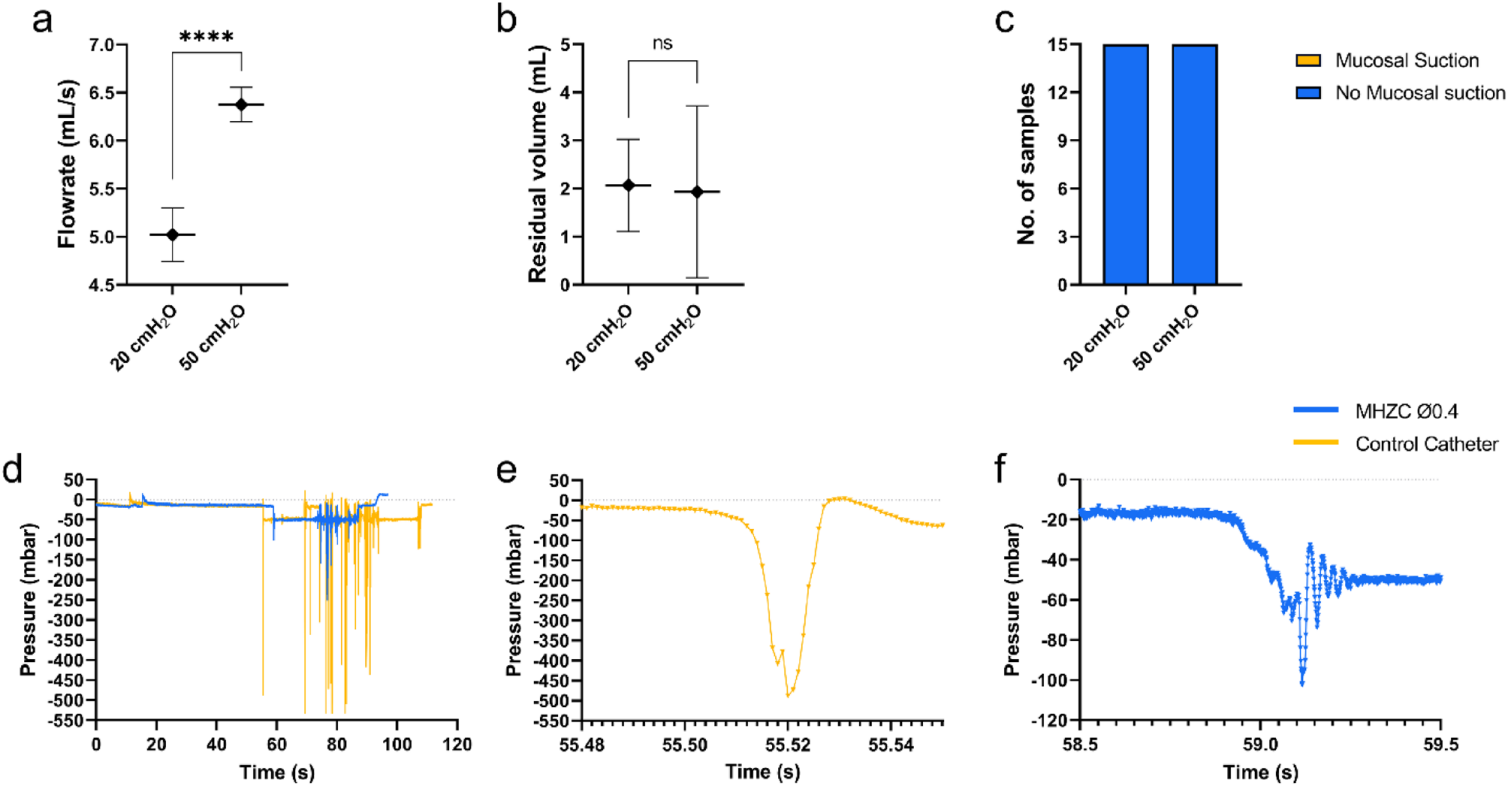
(**a**–**c**) Performance of MHZC with 0.4 mm diameter in ex vivo porcine lower urinary tract model at abdominal pressures chosen to mimic user sitting down (20 cmH_2_O) and standing up (50 cmH_2_O) scenarios, with panels showing (**a**) flowrate, (**b**) residual volume at the first flow-stop, and (**c**) instances of mucosal suction. (**d**) Overlay of typical pressure plots for CEC used as control (orange) and MHZC (blue) during catheterization, showing numerous sharp pressure peaks for the control and constant pressure for the MHZC catheter. (**e**) Detail of one pressure peak in the control catheter, with pressure variation close to 500 mbar and (**f**) detail of small pressure variation (below 100 mbar) with MHZC. ****p < 0.0001, CEC, conventional eyelet catheter; MHZC, micro-hole zone catheter.

Utilizing the MHZC with 0.4 mm hole diameter, the flowrate at 50 cmH_2_O was significantly higher than at 20 cmH_2_O (6.37 ± 0.18 mL/s vs 5.02 ± 0.28 mL/s, p < 0.0001, Fig. 3a). Similar differences at the 2 tested pressures were documented with the CEC^18^. Unlike the flowrates, the residual volumes were comparable, with average values around 2 mL at both pressures, as shown in Fig. 3b. In line with the results presented in Figs. 1c and 2c, no mucosal suction was documented, irrespective of the initial pressure (Fig. 3c).

In addition to measuring performance and safety outcomes as described above, a deeper understanding of the potential causes of these outcomes was gained by conducting intra-catheter pressure measurements at first flow stop. It was previously shown that mucosal suction events in CECs were associated with sudden variations in intra-catheter pressures, averaging around 300 mbar. Such pressure variations, perceived in a tactile way by the operator, were also correlated with flow-stops^18^. In the current study, pressure variations during catheterization using the MHZC were recorded at both 20 cmH_2_O and 50 cmH_2_O. Representative measurements are presented in Fig. 3d, showing an overlay of the pressure profiles, with clear differences between the two types of ICs. The CEC used as control was associated with pressure variations close to 500 mbar (example of an individual event is shown in Fig. 3e). In contrast to this, the MHZC showed a fairly constant pressure throughout the voiding process, with few and far smaller pressure peaks (Fig. 3d and example in Fig. 3f). A single instance of noticeable variation of close to 100 mbar was recorded in one draining experiment (detail in Fig. 3f), while no such variations were observed for any of the other experiments.

### Ex vivo and in vivo endoscopic examinations with the MHZC

**Figure 4:**
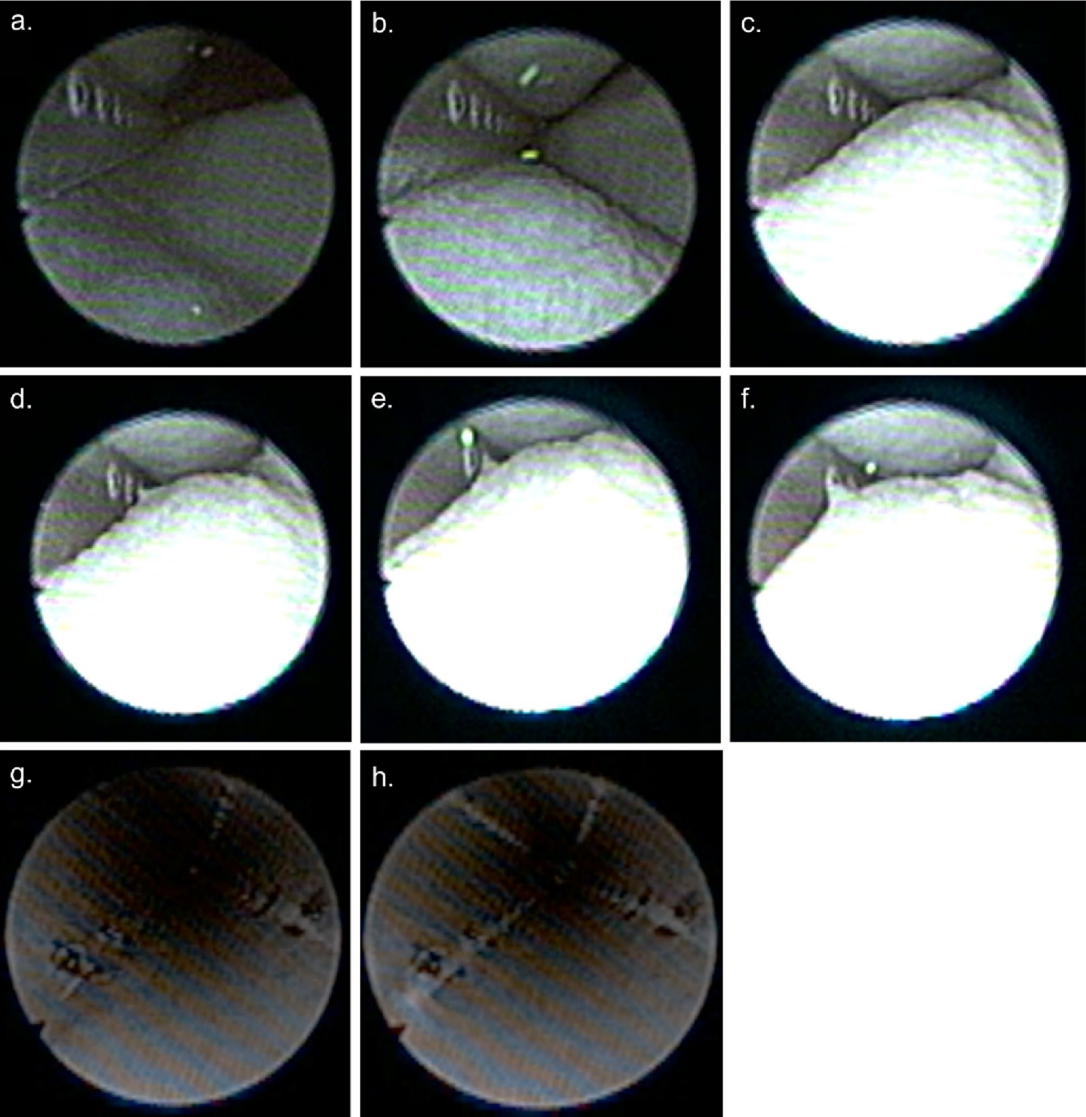
Endoscopic investigation of the ex vivo porcine lower urinary tract model during catheterization with MHZC with 0.4 mm diameter viewed from outside of the 12 CH catheter (**a**–**f**) or from the inside of the 16 CH catheter (**g**, **h**). (**a**) Beginning of the bladder voiding, followed by (**b**) the bladder starting to fold around the tip of the catheter as the voiding progresses, with fluorescent polyethylene microspheres being removed through the catheter. (**c**–**e**) As voiding continues, some of the 0.4 mm holes are sealed by the folded porcine bladder, but microspheres continue to be passed through the catheter, indicating flow is not blocked and even when (**f**) the last available micro-hole is partially closed by the folding bladder tissue, the from is still not completely interrupted. (**g**) View from inside the catheter during voiding and (**h**) view from inside the MHZC towards the end of the voiding process, with no mucosal suction observed through the micro-holes and into the catheter. MHZC, micro-hole zone catheter.

To gather further, more detailed understanding of how the MHZC with 0.4 mm hole diameter achieves the performance of very low residual volumes and increased flowrate with virtually no trauma-causing, flow-stopping mucosal suction events, catheterizations with parallel endoscopic examinations were carried out both ex vivo and in living pigs. A typical voiding process with the MHZC with 0.4 mm hole diameter in the ex vivo porcine LUT model is depicted as viewed from outside the catheter (Fig. 4a–f and Supporting Information) and from inside the device (Fig. 4g and h, as well as the video provided as supplementary material). The process, as observed from outside the IC (Fig. 4a–f), shows a gradual folding of the bladder around the tip of the catheter as the voiding progresses. While during bladder emptying the folding process eventually covers some of the micro-holes, complete blockage does not occur at any stage and voiding takes place with no interruptions. Towards the end of the catheterization, the bladder has enveloped most of the micro-holes, but, as seen in the views from inside the catheter, the bladder wall is not suctioned in through the micro-holes.

**Figure 5:**
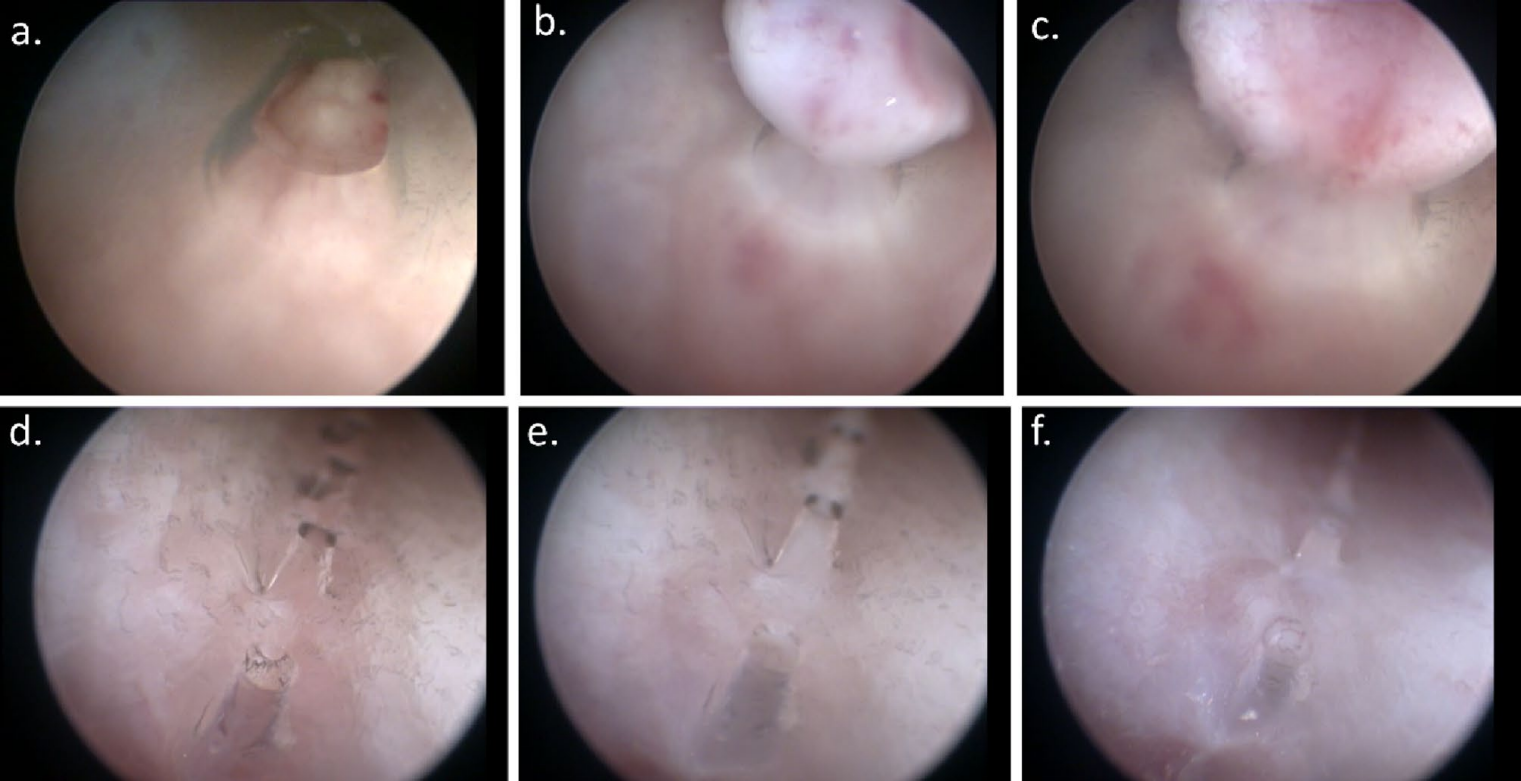
Endoscopic investigation in vivo, in female pig, showing the bladder emptying though a CEC (**a**–**c**) or through a CH16 MHZC with 0.4 mm hole diameter (**d**–**f**): (**a**–**c**) mucosal suction through the eyelet of a CEC, during bladder emptying, resulting in flow-stop (**d**) beginning of the voiding through the MHZC, (**e**) voiding progressions, through the MHZC and (**f**) voiding is temporarily interrupted, as bladder tissue can be seen entering inside the MHZC through some of the micro-holes, but complete mucosal suction and flow-stop do not occur. CEC, conventional eyelet catheter; MHZC, micro-hole zone catheter.

The behavior observed in the ex vivo model was further confirmed by in vivo endoscopic observations in live pigs. Figure 5 shows representative images from inside the MHZC during voiding, from beginning (Fig. 5a) to end (Fig. 5d). Flow-stops requiring catheter repositioning were not at any point detected in vivo using the MHZC, indicating that complete blocking of the micro-holes and mucosal suction did not occur.

## Discussion

Medical devices should ideally aim for the best performance and the highest levels of safety they can achieve. For bladder management through catheterization, this primarily means optimal bladder emptying and, at the same time, reducing adverse events such as mucosal microtrauma, which may lead to UTI^4,9,29,30^. While advancements have been made in recent years by improving catheter materials and coatings and moving towards single-use rather than reusable catheters^10,17,19,31,32^, other parameters and processes related to UTIs have not been studied as extensively. Many factors increase the patient's risk of developing a UTI when performing CIC; among them are incomplete or improper voiding reflected in high post-void residual urine volumes and mucosal microtrauma^10,11,33^.

As suggested by a recent ex vivo study, higher than desirable residual volumes may be explained by obstruction of flow caused by mucosal suction during voiding^18^. Specifically, a suction event and resulting flow-stop may be misinterpreted as the bladder being empty, thus leaving residual urine and potentially uropathogens in the bladder. Subsequently, repositioning of the catheter would be required to continue the voiding process. It has been documented that in practice CIC is sometimes associated with higher than ideal post-void residual volumes^11–14^.

The latter UTI contributing factor mentioned above, microtrauma, can result from mucosal suction through the catheter drainage holes, which may cause damage to the urothelial barrier^34^. In the case of CECs, the process leading to mucosal microtrauma is related to a hydrodynamically-generated negative-pressure gradient when the bladder tissue approaches and closes the eyelets, leading to negative pressure peaks at the site of the eyelets and subsequent transient suction of the urothelium^18,23,34^.

In order to tackle the 2 related issues described here, namely incomplete drainage and microtrauma due to suction events, a new IC has been developed. What differentiates it from a CEC is, primarily, the design of the drainage area. While in a typical IC the drainage area consists of 2 usually elongated, ellipse-like eyelets placed near the tip of the catheter, in the new MHZC these eyelets are replaced by over 80 micro-holes positioned in the same area where the eyelets would be in a conventional device. The micro-holes of the MHZCs tested in this study had diameters ranging from 0.4 to 0.7 mm whereas the ellipsoid-like eyelets on conventional catheters have sizes of around 4 mm (major axis) by 1.5 mm (minor axis)^34^.

The 2 types of ICs (MHZC and CEC) were tested in a previously developed ex vivo porcine LUT model. The CEC used as control here was the one that proved superior to other CECs in a previous study conducted in the same porcine LUT model^18^. The outcomes of interest reported in the current study were flowrate and residual volume at the first flow-stop, as indicators of catheter performance, as well as instances of mucosal suction related to potential safety issues, but also to risk of flow obturation. The impact of parameters such as diameter of micro-holes, catheter insertion depth, and simulated intra-abdominal pressure mimicking sitting and standing positions was analyzed in the study. However, irrespective of these parameters, the MHZCs outperformed the CEC in all of the experiments.

Two aspects are noteworthy, namely the very low residual volumes and the negligible mucosal suction attained with the MHZC. The residual volumes at the first flow-stop when using the MHZC were significantly lower than for the CEC in all cases, with values below 6 mL for the new device and typically around 40 mL when the CEC was used. This is indicative of the potential this new catheter design has to reduce the risk of UTI by decreasing the post-void residual urine volume to values that do not pose a threat for infection development^10,11^. From a different perspective, what this also means is that with the MHZC the first flow-stop occurred when all the urine from the bladder was drained and therefore was caused by the bladder being empty. By contrast, with the CEC the first flow-stop could take place, as previously shown^18^, not due to an empty bladder, but because of catheter obstruction, requiring subsequent repositioning of the device to complete the voiding.

Furthermore, the reduced incidence of mucosal suction episodes with the MHZC, or even lack thereof, with the 0.4 mm hole diameter, is suggestive of a different behavior of the device during voiding, compared to the CEC, where suction events were common (over 80% of catheterizations ex vivo). A deeper understanding of these differences was gained by measuring the pressure inside the catheter during bladder emptying and through endoscopic studies in the ex vivo model and also in vivo, in living pigs. As shown previously^18^ during voiding with a CEC the bladder mucosa may get suctioned into the eyelets and block the draining process. The endoscopy experiments conducted in the MHZC, however, show a different behavior, with the bladder gradually folding around the catheter tip and slowly covering the micro-holes, without obstructing flow. The lack of such suction events with the MHZC was further confirmed by the pressure profile inside the device during voiding, as a relatively constant pressure was recorded throughout the process. By contrast, the pressure profile of the CEC had sharp peaks, of up to 500 mbar, when suction events occurred, as shown previously^18^ and confirmed in this study.

A very recently published single-center clinical study used 42 male patients to test the MHZCs in comparison to a similar type of CEC^20^. Additionally, another new study on both male and female adults who were either healthy volunteers (n = 30) or intermittent catheter users (n = 30) compared the MHZC to a CEC in a single center, but conducting separate measurements on 3 different occasions^21^. These studies showed results close to the ex vivo data, with fewer flow-stop episodes and reduced residual urine using the MHZC compared to the CEC in both male and female patients^20,21^. The current study provides a detailed, in depth understanding of the MHZC’s functioning and performance under various conditions. It is important to note that employing the chosen ex vivo model allowed for conducting experiments that would not have been accessible in vivo, providing endoscopic visualization of the draining process. Furthermore, the use of the established porcine LUT model permitted a thorough examination into the influence of various device parameters (hole diameter) and user parameters (insertion depth, patient position during catheterization) that would be difficult to study in a clinical trial. This, once again, confirms the relevance of the ex vivo model.

The present investigation still, however, is limited by the fact that it does not assess the mucosal trauma directly, through histological examinations. A further limitation is related to having tested the MHZC in a model, reproducible setting, simulating only urine of normal density for this proof-of-concept work. As such, the results may not be entirely applicable to pyuria, or scenarios where the urine contains blood clots or other debris. Additional investigations are needed to fill these gaps that were beyond the scope of the current study. Nevertheless, the use of a validated porcine LUT model that closely resembles the human bladder is an important strength of the presented work. Furthermore, the previous use of the same model in the study of different CECs, together with similar results from the clinical trial^20^, further strengthens the validity of the results and provides the basis for future comparison to other tests conducted in the same ex vivo model.

The overarching conclusion of the current study is that the new catheter design, with over 80 micro-holes replacing the traditional 2 eyelets of conventional ICs, improves flowrates, reduces post-void residual volumes to values in the ideal range, and virtually eliminates suction events in a complex ex vivo bladder model. Furthermore, endoscopy analysis provided valuable insight that improved the understanding of how the voiding process of the MHZC differs from that of the conventional IC. By eliminating flow-stops resulting from eyelet obstruction, the need to reposition the catheter in practice is also eliminated, consequently reducing the risk of trauma inflicted by mechanically moving the device during catheterization. Collectively, the results of this study indicate a superior performance of the MHZC compared to the state-of-the-art CEC design on the key parameters of drainage efficiency and safeguarding of the bladder mucosa. Considering the association of these adverse effects with IC-related UTI, a lower risk of UTI when using this catheter is likely as well.

### Supplementary Information

Supplementary Legends.

Supplementary Video 1.

## Data Availability

The datasets used and/or analyzed during the current study available from the corresponding author on reasonable request.
