# Supplementary material for: New micro-hole zone catheter reduces residual urine and mucosal microtrauma in a lower urinary tract model
Source: Sci Rep. 2024 Jan 27;14:2268. doi: 10.1038/s41598-024-52505-6 (PMC10821950; doi:10.1038/s41598-024-52505-6)
Supplement: Supplementary file 1 — Supplementary Legends. [file 41598_2024_52505_MOESM1_ESM.docx]

**Supplementary material for:**

New micro-hole zone catheter reduces residual urine and mucosal microtrauma in a lower urinary tract model

Brit Schrødera, Fabio Tentora, Teodora Miclăușb, Kristian Stærkd,e, Thomas Emil Andersend,e, Michele Spinellif, Claudia Rendelig, Giulio Del Popoloh, Per Bagic, Lene Feldskov Nielsena,*

^a^ Coloplast A/S, Holtedam 1, 3050 Humlebaek, Denmark

^b^ AML Christensen, Nivaa Strandpark 21, Nivaa, Denmark

^c^ Rigshospitalet, Department of Urology, Blegdamsvej 9, 2100 Copenhagen, Denmark

^d^ Department of Clinical Microbiology, Odense University Hospital, Odense Denmark

^e^ Research Unit of Clinical Microbiology, University of Southern Denmark, Odense, Denmark

^f^ Spinal Unit, Niguarda Hospital, Milan, Italy

^g^ Department of Pediatrics, Università Cattolica del Sacro Cuore, Rome, Italy

^h^ Neuro-Urology Department, Azienda Ospedaliero-Universitaria Careggi, Florence, Italy

* Corresponding author: [dklfn@coloplast.com](mailto:dklfn@coloplast.com)

**1. Legend for the endoscope video**

**Video 1.** Endoscopic investigation in the ex-vivo porcine LUT model captured from the inside of a CH16 MHZC with 0.4 mm micro-hole diameter. A fiberoptic endoscope was placed inside the catheter in order to visualize the micro-holes during catheterization.
